# Water Vapor-Impermeable AlON/HfO*_x_* Bilayer Films Deposited by Hybrid High-Power Impulse Magnetron Sputtering/Radio-Frequency Magnetron Sputtering Processes

**DOI:** 10.3390/ma17225453

**Published:** 2024-11-08

**Authors:** Li-Chun Chang, Sheng-En Lin

**Affiliations:** 1 Department of Materials Engineering, Ming Chi University of Technology, New Taipei City 24301, Taiwan; m12188019@mail2.mcut.edu.tw; 2 Center for Plasma and Thin Film Technologies, Ming Chi University of Technology, New Taipei City 24301, Taiwan

**Keywords:** high-power impulse magnetron sputtering, thin-film encapsulation, water vapor transmission rate

## Abstract

Water vapor-impermeable AlON/HfO*_x_* bilayer films were constructed through a hybrid high-power impulse magnetron sputtering (HiPIMS) and radio-frequency magnetron sputtering process (RFMS), applied as an encapsulation of flexible electronics such as organic photovoltaics. The deposition of monolithic and amorphous AlON films through HiPIMS was investigated by varying the duty cycles from 5% to 20%. At an accelerated test condition, 60 °C, and 90% relative humidity, a 100 nm thick monolithic AlON film prepared using a duty cycle of 20% exhibited a low water vapor transmission rate (WVTR) of 0.0903 g m^−2^ day^−1^ after testing for 336 h. Furthermore, after introducing a nanocrystalline HfO*_x_* film through RFMS, a 214 nm thick AlON/HfO*_x_* bilayer film reached the lowest WVTR of 0.0126 g m^−2^ day^−1^.

## 1. Introduction

Flexible electronic devices have received much attention due to their prospective applications in rollup displays and flexible smart mobile devices. Polymer substrates are essential in flexible electronic devices, such as organic photovoltaics, thin-film transistors, and light-emitting diodes [1]. However, the severe water vapor permeability of polymer materials manifestly restricts the lifetime of flexible electronic devices. The familiar plastic substrates include polyethylene terephthalate (PET), polyimide, and polycarbonate. An essential concern for flexible electronics is their degradation, as they are susceptible to environmental moisture and oxygen, resulting in a short lifetime [2]. Therefore, constructing a thin-film encapsulation (TFE) layer has become a key technology for realizing flexible polymer devices. Various inorganic compounds, such as SiO*_x_* [3,4], Al_2_O_3_ [5], AlO*_x_*N*_y_* [6], TiO*_x_* [7,8], and SiN*_x_* [9], or their mixed oxides (Al_2_O_3_/HfO_2_ [10]) have been investigated as TFE layers. The processes for fabricating TFE layers need to be controlled at low temperatures due to the use of plastic substrates [10,11]. A low defect density is favored for preventing gas permeation [3,10]. Moreover, transparency to light is crucial for optoelectronic devices [3,10,11]. Al_2_O_3_ thin films with dense and defect-free structures fabricated by atomic layer deposition (ALD) have been developed as gas permeation barriers for organic light-emitting diode (OLED) devices, which show a water vapor transmission rate (WVTR) of 1 × 10^−3^ g m^−2^ day^−1^ when the film thickness is 26 nm and deposited on polyethylene naphthalate (PEN) substrates [12]. However, restrictions on the deposition temperatures and low deposition rates were the weaknesses of conventional ALD processes. The glass transition temperature (T_g_) of PEN is 126 °C. Though the ALD process performed in [12] was controlled at 120 °C, the deposition cycle was 60.25 s for the Al_2_O_3_ film, and the deposition rate was 0.12 nm/cycle. Moreover, plasma-assisted atomic layer deposition (ALD) has succeeded in developing moisture-impermeable Al_2_O_3_ barriers on PEN substrates using a low cycle of 17 s. It exhibits a WVTR of 5 × 10^−3^ g m^−2^ day^−1^ when the 20 nm thick film is deposited at room temperature [5]. Kim et al. [10] reported that 20 and 50 nm thick amorphous Al_2_O_3_ layers fabricated by plasma-enhanced ALD on PEN substrates had WVTRs of 2.92 × 10^−3^ and 3.26 × 10^−4^ g m^−2^ day^−1^, respectively, which increased by one order of magnitude when these films were grown at 100 °C. Similar increases in the WVTR with film thickness were obtained for the crystalline HfO_2_ and amorphous Al_2_O_3_/HfO_2_ mixed films. The 50 nm thick HfO_2_ film had a high WVTR of 6.75 × 10^−2^ g m^−2^ day^−1^, which was ascribed to its crystalline structure and provided moisture permeation pathways along grain boundaries and voids. In contrast, the 50 nm thick Al_2_O_3_/HfO_2_ films had a low WVTR of 1.44 × 10^−4^ g m^−2^ day^−1^.

The specifications of WVTR differ from those of various applications. The WVTR is 1–10 g m^−2^ day^−1^ for food packaging, 0.01 g m^−2^ day^−1^ for flexible electronic devices, and 10^−6^ g m^−2^ day^−1^ for the OLEDs [13]. The TFE layers for OLEDs with low moisture permeation are obtained through the ALD technique or inorganic and organic multilayer formations [14]. PET has been applied for versatile purposes, such as food storage, with a TFE layer for flexible device packaging [11]. Ascribing to the low T_g_ temperature of 66 °C for the amorphous PET, the utility of the expansive ALD process for raising moisture impermeability for PET is not worth developing. High-power impulse magnetron sputtering (HiPIMS) reveals the principal characteristics of the high ionization of the sputtered material [15], which resulted in the formation of densified films well adhered to the substrate and a droplet-free surface. HiPIMS technology is exemplified by low duty cycles, repetition frequency, and high power densities [16]. In this study, we prepared AlON films on PET substrates at room temperature through HiPIMS to improve the impermeability against water vapor and sustain high transparency. Moreover, HfO*_x_* films were reported to have high chemical stability [10]. Radio-frequency magnetron sputtering (RFMS) was widely applied to coat the oxide layers. A hybrid HiPIMS/RFMS was employed to advance the films’ quality and deposition rates [17]. Therefore, combining the benefits of AlON and HfO*_x_* films is a crucial attempt to develop TFE candidates. In this study, AlON/HfO*_x_* bilayer films were fabricated by hybrid HiPIMS/RFMS. The water vapor transmission rates (WVTRs) of the AlON/HfO*_x_* bilayer films were evaluated.

## 2. Materials and Methods

The monolithic AlON and HfO*_x_* films with a thickness of 97–110 nm and AlON/HfO*_x_* and HfO*_x_*/AlON bilayer films with a thickness of approximately 200 nm were fabricated on 50 μm thick PET sheets (BH216, Nan Ya Plastics, Taipei, Taiwan), glass slides (76 mm × 25 mm × 1 mm), and Si wafers. The substrate temperature was set at room temperature and heated by plasma to 43 °C during deposition, which did not exceed the T_g_ of 66 °C for the amorphous PET. The monolithic AlON films were prepared through HiPIMS using an Al target of 76.2 mm in diameter under an average power of 300 W. The samples D5, D10, D15, and D20 were prepared using on/off times of 50/950, 100/900, 150/850, and 200/800 μs in an HiPIMS cycle, respectively, representing a duty cycle of 5%, 10%, 15%, and 20% and a peak power density of 816, 481, 347, and 259 W/cm^2^, respectively. The introduced gas comprised 15 sccm Ar, 12 sccm N_2_, and 3 sccm O_2_, and the working pressure was maintained at 29 mPa. After depositing for 165 min, the thicknesses of samples D5, D10, D15, and D20 were 99, 97, 102, and 108 nm, respectively. The monolithic HfO*_x_* films were prepared using an HfO_2_ target of 76.2 mm diameter in the chamber purged with 30 sccm Ar. The thickness of HfO*_x_* films was 110 nm after depositing for 30 min. The AlON/HfO*_x_* and HfO*_x_*/AlON bilayer films with various thickness ratios were laminated with monolithic D20 and HfO*_x_* films by varying the deposition times.

The monolithic AlON and HfO*_x_* films’ chemical compositions were analyzed using a field-emission electron probe microanalyzer (FE-EPMA, JXA-iHP200F, JEOL, Tokyo, Japan) equipped with wavelength dispersive spectrometers at a 3 kV accelerating voltage. No signals from Si substrates were detectable. Al_2_O_3_, KAl_3_O_8_, BN, and Hf were the standard samples for evaluating the Al, O, N, and Hf compositions, respectively. The films’ phases were verified using X-ray diffraction (XRD; X’Pert PRO MPD, PANalytical, Almelo, The Netherlands) with Cu K_α_ radiation using a grazing incidence technique at an incidence angle of 1°. Film thicknesses were examined using a field-emission scanning electron microscope (FE-SEM, JSM-IT700HR, JEOL, Tokyo, Japan). The surface roughness values of the films were evaluated using atomic force microscopy (Dimension 3100 SPM, NanoScope IIIa, Veeco, NY, USA). Ra and Rq signify the average and root-mean-square surface roughness, respectively. The average values and standard deviations of surface roughness and chemical composition were determined from three measurements. The nanostructures of the films were observed using transmission electron microscopy (TEM, JEM-2010E, JEOL, Akishima, Japan). The TEM samples with a protective C or Pt layer were prepared using a focused ion beam system (NX2000, Hitachi, Tokyo, Japan). Optical transmission was examined using a spectrophotometer (v-650, Jasco, Tokyo, Japan).

**Figure 1:**
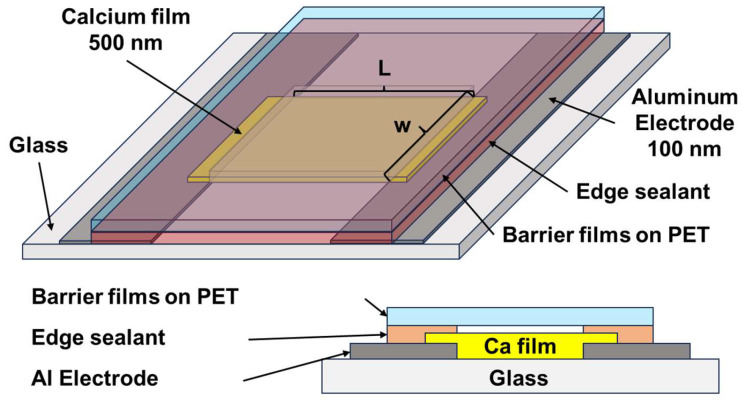
Structure of the calcium test for WVTR measurement.

The WVTRs of AlON films were tested using a water vapor permeation analyzer (Aquatran Model 2, MOCON, Brooklyn Park, MN, USA) conducted at 60 °C in 90% relative humidity for 24 h. The MOCON instrument and calcium test are commonly used to determine films’ WVTR [9,18]. Moreover, a homemade calcium test system evaluated the WVTRs of AlON films and AlON/HfO*_x_* bilayer films at 60 °C in 90% relative humidity for 336 h. Figure 1 displays the sample structure for the calcium test in this study. The WVTR is determined as follows [10,18,19]:

(1) $$WVTR=n\delta_{Ca}\rho_{Ca}\left(\frac{L_{eff}}{w}\right)\left(\frac{A_{Ca}}{A_{B}}\right)\left(\frac{M_{H_{2}O}}{M_{Ca}}\right)\left(\frac{d\left(1/R\right)}{dt}\right),$$

where *n* is the reaction ratio between water and Ca (2), *δ*_Ca_ is the density of Ca (1.55 g/cm^3^), *ρ*_Ca_ is the resistivity of Ca film (3.9 μΩcm), *L_eff_* (1 cm) and *w* (0.5 cm) are, respectively, the effective length and width of the Ca test lines, *A*_Ca_/*A_B_* is the ratio of the Ca test sensor area to the H_2_O vapor barrier area (1), *M*_H_2_O_/*M*_Ca_ is the molecular weight ratio of H_2_O to Ca (18/40), *d*(1/*R*)/*dt* is the variation in conductivity with time *t*, and *R* is the resistivity. Three measurements were made to determine the WVTR values.

## 3. Results and Discussion

### 3.1. Monolithic AlON and HfO_x_ Films

Table 1 shows the atomic compositions of the monolithic AlON films prepared by HiPIMS processes. The AlON films prepared using a duty cycle of 5% exhibited an atomic composition of 38.54% Al–59.31% O–2.15% N. The sputter gas flow consisted of 15 sccm Ar, 12 sccm N_2_, and 3 sccm O_2_. O and Al’s affinity was higher than N and Al’s. Moreover, the AlON films prepared using higher duty cycles of 10%, 15%, and 20% exhibited slightly higher O and lower Al and N contents. The stoichiometric ratios of anions to cations in Al_2_O_3_ and AlN compounds were 1.5 and 1, respectively. The required Al content to achieve stoichiometric Al_2_O_3_ and AlN, labeled as Al*, was higher than the realistic Al content. The Al*/Al ratios were 1.08, 1.14, 1.13, and 1.16 for D5, D10, D15, and D20 films, respectively, which implies that all of the fabricated monolithic AlON films were over-stoichiometric with extra O and N contents.

**Figure 2:**
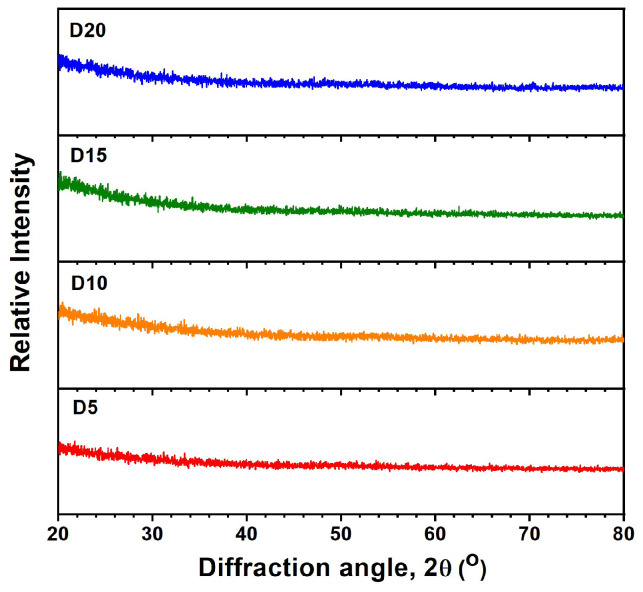
XRD patterns of monolithic AlON films prepared using various duty cycles in HiPIMS processes.

**Figure 3:**
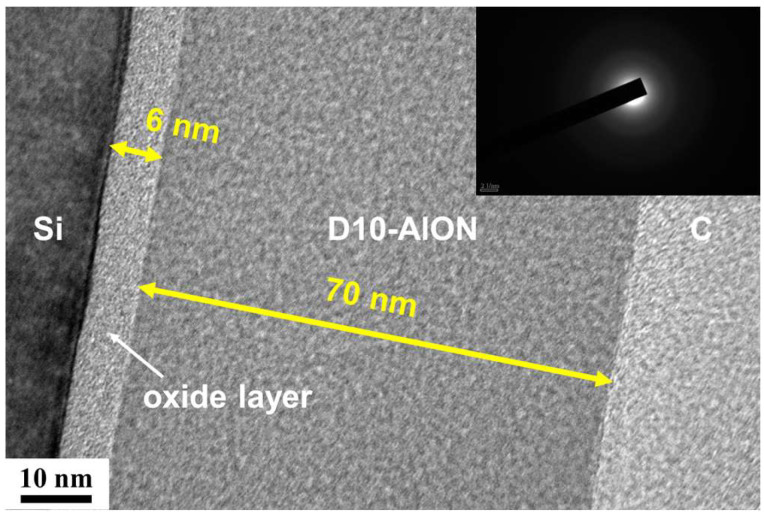
Cross-sectional TEM image of the D10-AlON film.

**Figure 4:**
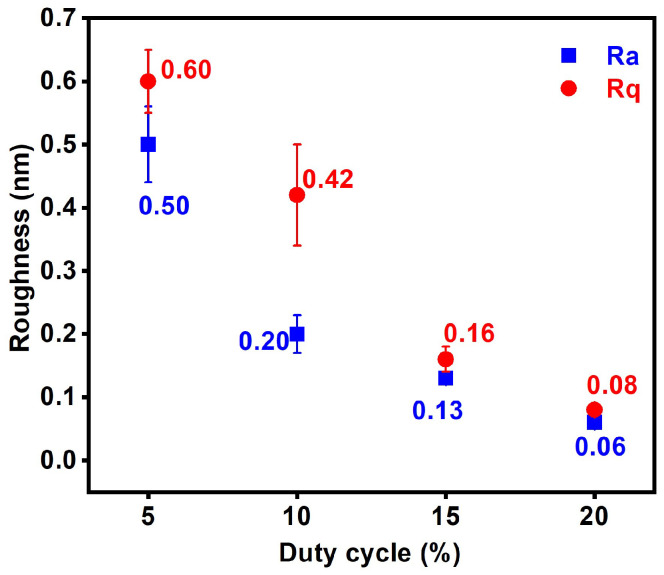
Surface roughness values of monolithic AlON films prepared on Si substrate.

**Figure 5:**
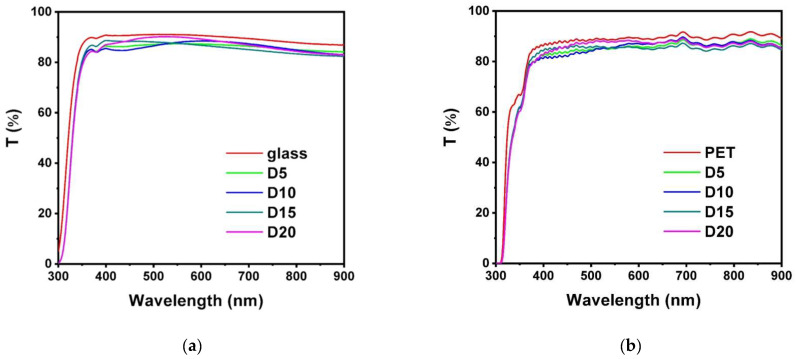
Transmission of monolithic AlON films prepared on (**a**) glass and (**b**) PET.

Figure 2 depicts the XRD patterns of the monolithic AlON films. All of the AlON films exhibited amorphous structures. Figure 3 displays a cross-sectional TEM image of the D10-AlON film prepared on a Si substrate. An oxide layer that was 6 nm thick formed on the Si substrate, and the D10 film exhibited an amorphous structure. The oxide layer consisted of native oxide and followed the diffusion of Si and O in the subsequent AlON deposition process. The selective area electron diffusion (SAED) pattern without clear spots or rings confirms the amorphous phase formation. Figure 4 shows the Ra and Rq values of the monolithic AlON films, which decreased from 0.50 to 0.06 nm and 0.60 to 0.08 nm, with duty cycles increasing from 5% to 20%. All of the Ra and Rq values were less than 0.6 nm, which was attributed to the formation of amorphous structures. Figure 5 exhibits the optical transmittance of the AlON films prepared on glass and PET substrates, respectively. The transmittance ratios at 530 nm of the glass substrate and the D5, D10, D15, and D20 films prepared on glass substrates were 91.0%, 87.3%, 87.5%, 87.8%, and 90.2%, respectively. In contrast, the transmittance ratios of the PET substrate and the D5, D10, D15, and D20 films prepared on PET substrates were 88.8%, 85.1%, 85.3%, 85.7%, and 87.9%, respectively. The transmittance ratios for the samples prepared on PET substrates were lower than those prepared on glass substrates, which were attributed to the difference between the transmittance ratios of the substrates. All of the roughness values of the films deposited on glass and PET substrates were <0.6 nm. The wavelength of visible light is far larger than the roughness of the samples; therefore, the influence of roughness on optical transmission can be negligible.

**Figure 6:**
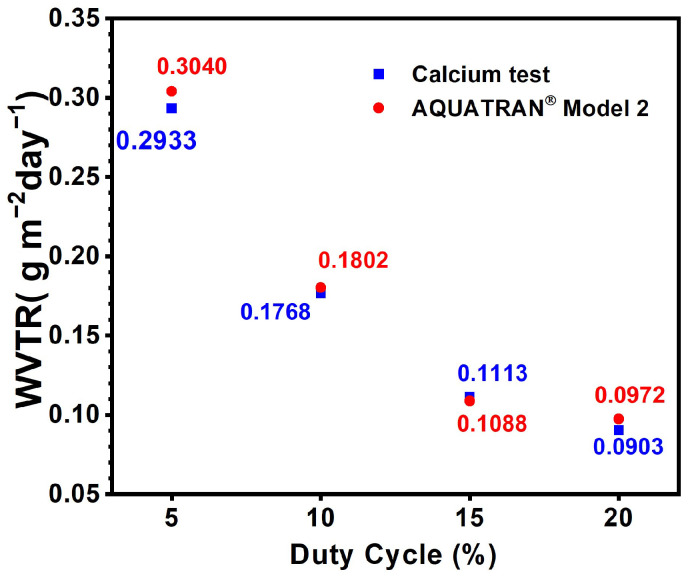
WVTRs of the monolithic AlON films prepared on PET substrates and examined by a water vapor permeation analyzer for 24 h and calcium test for 336 h at 60 °C in 90% relative humidity.

Figure 6 displays the WVTRs of the monolithic AlON films prepared on PET substrates. The WVTR analyzed using the water vapor permeation analyzer exhibited a decreasing trend from 0.3040 to 0.1802, 0.1088, and 0.0972 g m^−2^ day^−1^ when the duty cycle for fabricating AlON films increased from 5% to 10%, 15%, and 20%. The WVTR evaluated by the calcium test decreased from 0.2933 to 0.1768, 0.1113, and 0.0903 g m^−2^ day^−1^ when the duty cycle increased from 5% to 10%, 15%, and 20%, which exhibited a highly consistent trend relative to that examined by the water vapor permeation analyzer. The increase in the duty cycle of the HiPIMS process resulted in a decrease in the impact energy of the sputtered atoms and decreased defect formation, which reduced the diffusion paths of the moisture and the WVTR.

**Figure 7:**
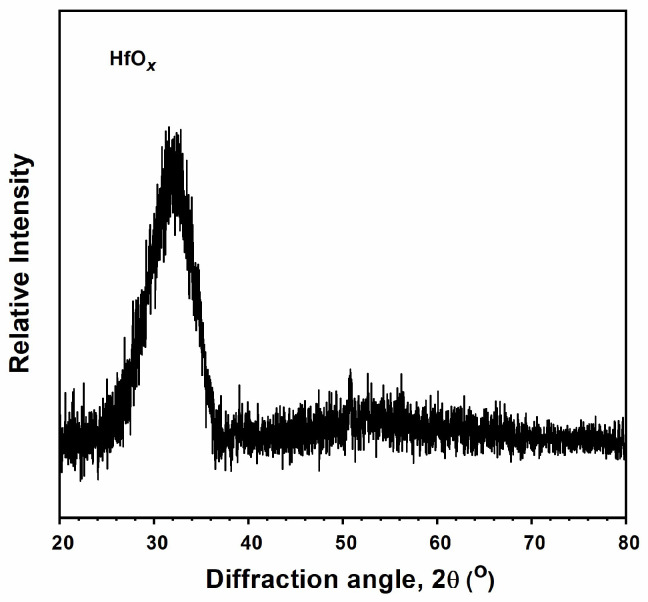
XRD patterns of monolithic HfO*_x_* films.

The atomic composition of the monolithic HfO*_x_* films prepared through RFMS was 40.53 ± 0.18% Hf and 59.47 ± 0.18% O. The stoichiometric ratio of O/Hf was 1.47 (<2), which implied that the monolithic HfO*_x_* films were under-stoichiometric. Figure 7 depicts the XRD pattern of the monolithic HfO*_x_* films, revealing a nanocrystalline structure with a broad reflection at around a 2θ angle of 32°.

### 3.2. AlON/HfO_x_ Bilayer Films

**Figure 8:**
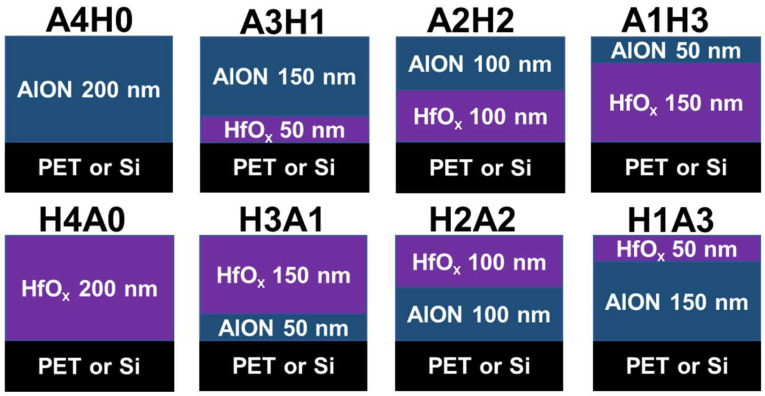
Schemes of the bilayer films.

**Figure 9:**
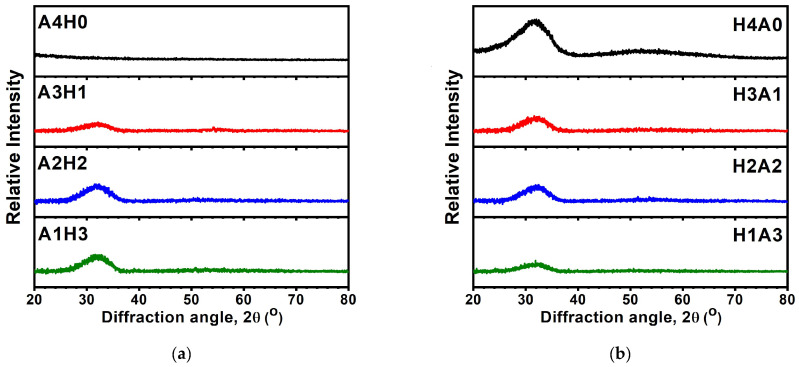
XRD patterns of (**a**) A4H0, A3H1, A2H2, and A1H3 and (**b**) H4A0, H3A1, H2A2, and H1A3 bilayer films.

**Figure 10:**
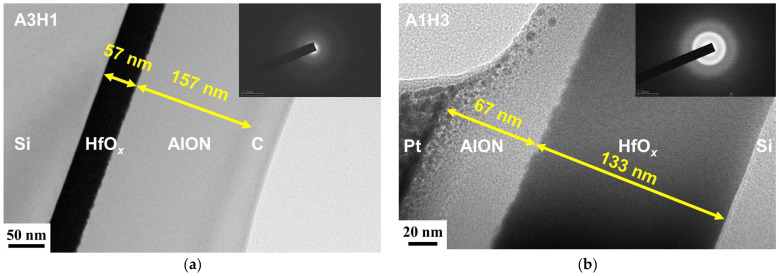
Cross-sectional TEM and SAED patterns of (**a**) A3H1 and (**b**) A1H3 bilayer films.

Figure 8 displays the design of the AlON/HfO*_x_* and HfO*_x_*/AlON bilayer films. The monolithic AlON and HfO*_x_* films with a higher thickness of approximately 200 nm were also prepared for comparison and renamed A4H0 and H4A0, respectively. According to the deposition rates of the monolithic D20 and HfO*_x_* samples, the bilayer films were regulated by various deposition times. For example, the A3H1 bilayer films were deposited using the condition for fabricating HfO*_x_* for 10 min and then deposited using the condition for preparing AlON(D20) for 120 min. The A2H2 bilayer films were deposited on an HfO*_x_* sublayer for 20 min and an AlON(D20) sublayer for 80 min. The A1H3 bilayer films were deposited on an HfO*_x_* sublayer for 30 min and an AlON(D20) sublayer for 40 min. Figure 9 displays the XRD patterns of the AlON/HfO*_x_* and HfO*_x_*/AlON bilayer films. The A4H0 film, a thicker monolithic D20 film, exhibited amorphous structures. In contrast, the A3H1, A2H2, and A1H3 AlON/HfO*_x_* bilayer films revealed nanocrystalline structures, ascribed to the HfO*_x_* sublayers. Figure 10 depicts cross-sectional TEM images of the A3H1 and A1H3 films. The A3H1 film is stacked with a 57 nm thick HfO*_x_* sublayer and a 157 nm thick AlON sublayer. The SAED pattern correlated to the AlON sublayer exhibits amorphous structures. The A1H3 film is stacked with a 133 nm thick HfO*_x_* sublayer and a 67 nm thick AlON sublayer. The SAED pattern correlated to the HfO*_x_* sublayer reveals a nanocrystalline structure.

**Figure 11:**
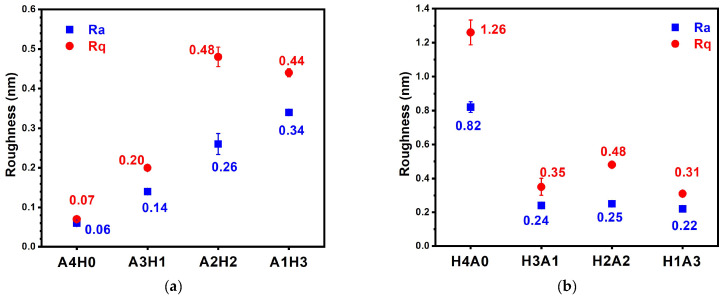
Surface roughness values of (**a**) A4H0, A3H1, A2H2, and A1H3 and (**b**) H4A0, H3A1, H2A2, and H1A3 bilayer films.

Figure 11 shows the surface roughness values of the AlON/HfO*_x_* and HfO*_x_*/AlON bilayer films. All of the bilayer films exhibited Ra and Rq values of less than 0.5 nm. The monolithic A4H0 films exhibited Ra and Rq values of <0.1 nm, which was attributed to the amorphous structure, as observed from the XRD pattern of the A4H0 film and the SAED pattern of the A3H1 film. Combined with amorphous AlON sublayers, the AlON/HfO*_x_* and HfO*_x_*/AlON bilayer films revealed low surface roughness values. In contrast, the monolithic H4A0 film exhibited high Ra and Rq values of 0.82 and 1.26 nm, respectively. The low surface roughness values of the bilayer films were dominated by the presence of an AlON sublayer either in the top or bottom layers.

**Figure 12:**
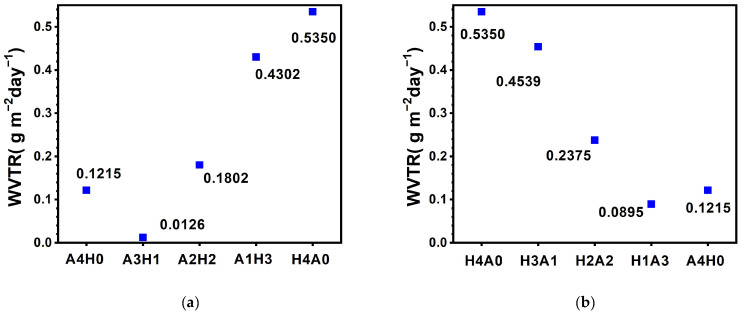
WVTRs of (**a**) A4H0, A3H1, A2H2, and A1H3 AlON/HfO*_x_* bilayer films and (**b**) H4A0, H3A1, H2A2, and H1A3 HfO*_x_*/AlON bilayer films after testing at 60 °C in 90% RH for 336 h.

Figure 12 exhibits the WVTRs of the AlON/HfO*_x_* and HfO*_x_*/AlON bilayer films examined by the calcium test at 60 °C in 90% RH for 336 h. The monolithic A4H0 film with a designed thickness of 200 nm showed a WVTR of 0.1215 g m^−2^ day^−1^, which was slightly higher than the 0.0903 g m^−2^ day^−1^ of the D20-AlON film with a thickness of 108 nm. Chen et al. [11] reported chemical vapor-deposited diamond-like carbon films with a thickness of 793 nm on PET substrates (47 μm thick), revealing a WVRT of 0.12 g m^−2^ day^−1^ at 25 °C in 80% RH. Lee et al. [20] reported plasma-enhanced chemical vapor-deposited SiN*_x_* and SiO*_x_* films (200 nm thick) on PET (100 μm thick), exhibiting WVRTs of 0.03 and 0.06 g m^−2^ day^−1^ at 60 °C in 90% RH after 10 days of testing. The A3H1 bilayer film has the thickest AlON top sublayer and exhibits the lowest WVTR among the AlON/HfO*_x_* bilayer films at 0.0126 g m^−2^ day^−1^. With the decreasing thickness of the AlON top layer, the A2H2 bilayer film exhibits a higher WVTR of 01802 g m^−2^ day^−1^. Moreover, the A1H3 bilayer film with a successively decreased AlON thickness has a much higher WVTR of 0.4302 g m^−2^ day^−1^. However, this is lower than 0.5350 g m^−2^ day^−1^ for the monolithic H4A0 film. The WVTR of the H4A0 film is 4.4 times greater than that of the A4H0 film. The bilayer film with a higher AlON thickness ratio exhibits a lower WVTR. In contrast, the A3H1 film has a WVTR lower than 0.1215 g m^−2^ day^−1^ of the A4H0 film, ascribed to the chemically stable HfO*_x_* sublayer [10]. Moreover, the formation of a hetero-interface between the AlON and HfO*_x_* sublayers could play a vital role in restricting moisture permeation. The effect of the heterointerface will be investigated in a future study by evaluating the WVTRs of multilayers with various stacking periods. On the other hand, the HfO*_x_*/AlON bilayer films exhibited a similar trend in WVTR with the AlON sublayer’s thickness variation.

## 4. Conclusions

Monolithic AlON film and AlON/HfO*_x_* and HfO*_x_*/AlON bilayer films were fabricated through hybrid HiPIMS/RFMS processes to evaluate their applications as water vapor-impermeable films on PET. The AlON and HfO*_x_* films were amorphous and nanocrystalline, respectively. The WVTR evaluated using a calcium test system revealed highly reliable results that agreed with those examined by the water vapor permeation analyzer. The AlON films exhibited a lower WVTR than that of the HfO*_x_* films. Moreover, the AlON/HfO*_x_* and HfO*_x_*/AlON bilayer films with higher thickness ratios of AlON/HfO*_x_* have lower WVTRs than the monolithic AlON films. The lowest WVTR of 0.0126 g m^−2^ day^−1^ was obtained for 214 nm thick AlON/HfO*_x_* bilayer films after testing at 60 °C under 90% relative humidity for 336 h.

## Figures and Tables

**Table 1:** Atomic compositions of AlON films.

Sample	Duty Cycle	Atomic Compositions (at.%)		
		Al	O	N
D5	5%	38.54 ± 0.33	59.31 ± 0.30	2.15 ± 0.25
D10	10%	37.13 ± 0.55	61.10 ± 0.54	1.77 ± 0.15
D15	15%	37.51 ± 0.27	60.61 ± 0.27	1.88 ± 0.12
D20	20%	36.89 ± 0.41	61.26 ± 0.33	1.85 ± 0.20

## Data Availability

Data are contained within the article.
